# Immune Checkpoint Dysregulation in Aneurysmal Subarachnoid Hemorrhage: A Prospective Study of sCTLA-4 and sPD-L1 as Biomarkers of Symptomatic Vasospasm

**DOI:** 10.3390/ijms26178228

**Published:** 2025-08-25

**Authors:** Reka Varnai, Gábor J. Szebeni, Nikolett Gémes, Attila Schwarcz, Tihamer Molnar, Csaba Olah, Peter Csecsei

**Affiliations:** 1 Department of Primary Health Care, Medical School, University of Pecs, 7622 Pecs, Hungary; varnai.reka@pte.hu; 2 Laboratory of Flow Cytometry, Core Facility, HUN-REN Biological Research Centre, 6726 Szeged, Hungary; szebeni.gabor@brc.hu (G.J.S.); gemes.nikolett@brc.hu (N.G.); 3 Department of Internal Medicine, Hematology Centre, Faculty of Medicine, University of Szeged, 6725 Szeged, Hungary; 4 Department of Neurosurgery, Medical School, University of Pecs, 7622 Pecs, Hungary; schwarcz.attila@pte.hu; 5 Department of Anaesthesiology and Intensive Care, Medical School, University of Pecs, 7622 Pecs, Hungary; molnar.tihamer@pte.hu; 6 Department of Neurosurgery, Borsod-Abaúj-Zemplén County Center Hospital and University Teaching Hospital, 3526 Miskolc, Hungary; olahcs@gmail.com

**Keywords:** aneurysmal subarachnoid hemorrhage, CTLA-4, PD-L1, symptomatic cerebral vasospasm

## Abstract

Aneurysmal subarachnoid hemorrhage (aSAH) is a severe stroke subtype often complicated by symptomatic cerebral vasospasm (sVP), contributing to delayed cerebral ischemia and poor outcomes. Immune dysregulation, particularly T-cell imbalances and pro-inflammatory cytokines, is implicated in vasospasm development. Soluble immune checkpoint proteins—CTLA-4 (sCTLA-4) and PD-L1 (sPD-L1)—regulate immune homeostasis and may serve as biomarkers or modulators of inflammation in aSAH. This prospective cohort study included 179 aSAH patients, divided into sVP+ (*n* = 48) and sVP− (*n* = 131), plus 50 healthy controls. Serum sCTLA-4 and sPD-L1 levels were measured on days 1, 5, and 9 post-ictus using Luminex xMAP. Associations with clinical outcomes were analyzed using non-parametric statistics and hierarchical clustering. Both sCTLA-4 and sPD-L1 were significantly elevated in sVP+ patients versus sVP− and controls, increasing over time. sCTLA-4 was significantly higher in sVP+ on days 5 (*p* = 0.001) and 9 (*p* < 0.001), and sPD-L1 on days 5 and 9 (both *p* < 0.001). Clustering revealed distinct expression patterns between sVP+ and sVP− groups. Elevated sCTLA-4 and sPD-L1 levels are associated with sVP after aSAH and may serve as biomarkers for early immune dysfunction, offering insights into potential therapeutic targets.

## 1. Introduction

Aneurysmal subarachnoid hemorrhage (aSAH) is a life-threatening condition accounting for 5–10% of all strokes, with high morbidity and a mortality rate of up to 50% despite advances in treatment [1]. Cerebral vasospasm typically develops between 3 and 14 days following an aneurysmal subarachnoid hemorrhage (aSAH) and represents the leading cause of delayed morbidity and mortality. Angiographic evidence of vasospasm in the cerebral arteries is observed in up to 70% of patients within the first two weeks after an intracranial aneurysm (IA) rupture, with 20–40% experiencing symptomatic vasospasm resulting in clinical decline or neurologic deficits due to delayed cerebral ischemia (DCI) [2,3].

Immune dysfunction plays a central role in the development of cerebral vasospasm after aSAH by driving a sustained pro-inflammatory response in the central nervous system. This involves early infiltration of neutrophils, activation of microglia, and an imbalance between pro-inflammatory Th17 cells and regulatory T cells, which collectively promote vascular inflammation, endothelial damage, and arterial narrowing leading to vasospasm [4]. CTLA-4 (cytotoxic T-lymphocyte antigen-4) and PD-L1 (programmed death ligand 1) are immune checkpoint molecules that act as negative regulators of the immune system to maintain self-tolerance and prevent overactivation [5,6].

Soluble CTLA-4 (sCTLA-4) is a splice variant of the membrane-bound CTLA-4 receptor, lacking the transmembrane domain and thus secreted into the bloodstream [5]. It plays a significant role in modulating immune responses by binding to B7 ligands (CD80/CD86) on antigen-presenting cells, thereby inhibiting T-cell activation [7]. sCTLA-4 appears at low levels in serum following CD4^+^ T cell activation, indicating its limited but detectable secretion into circulation [8]. sCTLA-4 participates in immune regulation by potentiating the function of Treg cells [9], and sCTLA-4 is essential for immune homeostasis and controlling type 1 immunity while allowing type 2 immunity to facilitate resolution in inflammatory conditions [10]. In stroke and other cerebrovascular conditions, impaired CTLA-4 expression can lead to excessive inflammation, while CTLA-4 activation via regulatory T cells supports neuroprotection and offers a promising target for immunomodulatory therapies [11].

Soluble PD-L1 (sPD-L1) is a circulating form of the PD-L1 immune checkpoint protein that plays an important immunoregulatory role in various pathological and physiological contexts. It exists in multiple forms [12], generated either by proteolytic cleavage of membrane-bound PD-L1 (mPD-L1) and exosomal PD-L1 (exoPD-L1) by enzymes such as MMPs [13] and ADAM proteases [14], or through alternative splicing of PD-L1 pre-mRNA to produce isoforms lacking the transmembrane domain [15,16]. These forms retain the ability to bind PD-1 and often suppress T-cell activity [17], thereby contributing to immune evasion in cancer and immunosuppression in inflammatory and viral diseases [18]. sPD-L1 is detectable in blood [19] and has been associated with worse prognosis in many cancers [20], although its predictive value varies depending on tumor type. Additionally, sPD-L1 levels are elevated in numerous non-malignant conditions such as autoimmune diseases [21,22] or sepsis [23], often reflecting a general marker of inflammation. In this study, serum sPD-L1 was quantified using a multiplex immunoassay that detects total soluble PD-L1 without discriminating between isoforms derived from proteolytic cleavage [13,14] or alternative splicing. We specifically selected sPD-L1 because the PD-1/PD-L1 pathway is a central immune checkpoint axis with relevance to both systemic inflammation and cerebrovascular injury.

Dysregulated expression or function of CTLA-4 or PD-L1 could exacerbate the Th17/Treg imbalance, leading to unchecked pro-inflammatory T-cell activation, microglial activation, and vascular inflammation. In the early phase of aSAH, where immune activation is key (e.g., high IL-6, TNF-α, and IL-17), a lack of immune checkpoint signaling may fail to restrain excessive inflammation that contributes to symptomatic vasospasm.

Symptomatic cerebral vasospasm following aSAH involves a pro-inflammatory immune response, particularly characterized by T-cell imbalance and heightened cytokine activity [4]. Since sPD-L1 and sCTLA-4 can modulate systemic and local inflammation, their dysregulation may contribute to or reflect the immune dysfunction underlying vasospasm. Additionally, their presence in circulation makes them accessible biomarkers for studying immune activity in this condition.

The aim of our study was to investigate the role of sCTLA-4 and sPD-L1 in the development of symptomatic vasospasm.

## 2. Results

### 2.1. Study Population

Between January 2022 and December 2024, a total of 225 patients with aneurysmal subarachnoid hemorrhage (aSAH) were screened for eligibility. Of these, 46 patients were excluded based on predefined criteria: six due to active or recent malignancy, six with autoimmune or immunological diseases, three with chronic renal failure, and two with chronic hepatic failure. Additional exclusions included eight patients with incomplete biomarker sampling, six who declined informed consent, two with aneurysm rerupture during treatment, three admitted more than 24 h after symptom onset, two presenting with active infection at admission, and one with an arteriovenous malformation (AVM) as the bleeding source. Seven patients were lost to follow-up and excluded from the final analysis. The remaining 179 patients were included in the study and underwent full biomarker assessment and clinical follow-up.

The study population data compares two groups: patients without symptomatic vasospasm (sVP−, *n* = 131) and those with symptomatic vasospasm (sVP+, *n* = 48). The average age was similar across groups (58 vs. 56 years; *p* = 0.374), and gender distribution was also comparable (71% vs. 76% female; *p* = 0.597). Common comorbidities such as hypertension (51% vs. 58%; *p* = 0.432), diabetes (8% vs. 13%; *p* = 0.425), and smoking (30% vs. 27%; *p* = 0.863) showed no significant differences. Neurological severity markers such as WFNS score (median 2 in both groups) and modified Fisher score (median 3) were evenly distributed, with *p*-values of 0.673 and 0.290, respectively. Clinical events at presentation—loss of consciousness and seizures—were equally common across groups (38% and 9–11%, respectively; *p*-values > 0.5). Aneurysm locations were broadly distributed, with the anterior communicating artery being most frequent, and no significant group differences observed. Admission laboratory markers, including hsCRP, creatinine, white blood cell count, and neutrophil-lymphocyte ratio, had overlapping medians and wide IQRs, with non-significant *p*-values (all > 0.1).

Procedural interventions such as lumbar drainage, mechanical ventilation, decompressive craniotomy, and extraventricular drainage did not differ significantly between groups, although lumbar drain use approached significance (47% vs. 62%; *p* = 0.083). The only statistically significant difference was in infection rates: 46% in the sVP+ group versus 26% in the sVP− group (*p* = 0.011). The detailed characteristics of the study population can be found in Table 1.

### 2.2. Serum CTLA-4 and PD-L1 Levels in sVP− and sVP+ Groups

**Figure 1:**
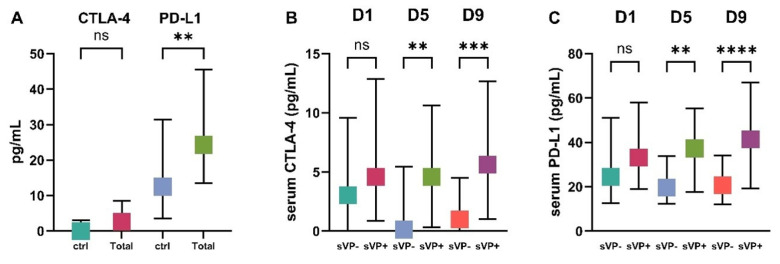
Serum levels (pg/mL) of immune checkpoint molecules CTLA-4 and PD-L1 in patients with aneurysmal subarachnoid hemorrhage (aSAH) with (sVP+) and without (sVP−) symptomatic vasospasm (sVP) on day 1 (D1), day 5 (D5), and day 9 (D9) after ictus. (**A**) Comparison of CTLA-4 and PD-L1 serum concentrations between healthy controls (ctrl) and the total aSAH patient population (Total). (**B**) Temporal changes in serum CTLA-4 levels in patients with (sVP+) and without (sVP−) secondary vasospasm on days 1 (D1), 5 (D5), and 9 (D9) post-ictus. (**C**) Temporal changes in serum PD-L1 levels in sVP− and sVP+ patients on D1, D5, and D9. Statistical analysis was performed using the Mann–Whitney U test. Data are presented as medians with interquartile ranges. CTLA-4, cytotoxic T-lymphocyte-associated protein 4; PD-L1, programmed death-ligand 1; sVP, symptomatic vasospasm; ctrl, control; D1/D5/D9, days 1, 5, and 9 post-ictus; ns, not significant. **** *p* < 0.0001; *** *p* < 0.001; ** *p* < 0.01.

Figure 1 and Table 2 illustrate the temporal dynamics and intergroup differences in serum levels of CTLA-4 and PD-L1. On day 1 (D1), median CTLA-4 levels were slightly elevated in the sVP+ group (4.6 pg/mL, IQR 1–12.5) compared to the sVP− group (3 pg/mL, IQR 0–9.6), although this difference was not statistically significant. However, CTLA-4 levels in sVP+ patients were significantly higher than those in controls (0 pg/mL, IQR 0–3.1; *p* = 0.002). By day 5, CTLA-4 levels in the sVP+ group remained elevated (median 4.6 pg/mL), and a significant difference emerged between sVP+ and sVP− patients (*p* = 0.001). This pattern persisted on day 9, with sVP+ patients showing the highest CTLA-4 levels (median 5.6 pg/mL, IQR 1–12.7), significantly higher than both sVP− patients (1 pg/mL, IQR 0–4.5; *p* < 0.001) and controls (*p* < 0.001). There was no significant difference in CTLA-4 levels between sVP− patients and controls on any day.

For PD-L1, median serum levels were consistently higher in the sVP+ group across all time points. On D1, the sVP+ group showed a median PD-L1 level of 33.2 pg/mL (IQR 19.4–54.2), compared to 24.5 pg/mL (IQR 12.7–51.1) in the sVP− group and 12.5 pg/mL (IQR 3.6–31.5) in controls. Although the sVP+ vs. sVP− difference was not statistically significant on D1, both sVP− (*p* = 0.006) and sVP+ (*p* < 0.001) groups exhibited significantly elevated PD-L1 compared to controls. On D5 and D9, PD-L1 levels in the sVP+ group continued to rise (D5: 37.3 pg/mL; D9: 41.4 pg/mL), maintaining significant differences compared to sVP− (D5: 19.7 pg/mL, *p* = 0.001; D9: 20.7 pg/mL, *p* < 0.001) and controls (both *p* < 0.001). There were no significant differences between sVP− and controls on D5 or D9.

In the vasospasm-positive (sVP+) group, CTLA-4 and PD-L1 levels remain consistently elevated regardless of the presence or absence of infection, with no meaningful differences between infected and non-infected subgroups at either D5 or D9, Table 3.

### 2.3. Hierarchical Clustering of Serum CTLA-4 and PD-L1 Levels over Time

**Figure 2:**
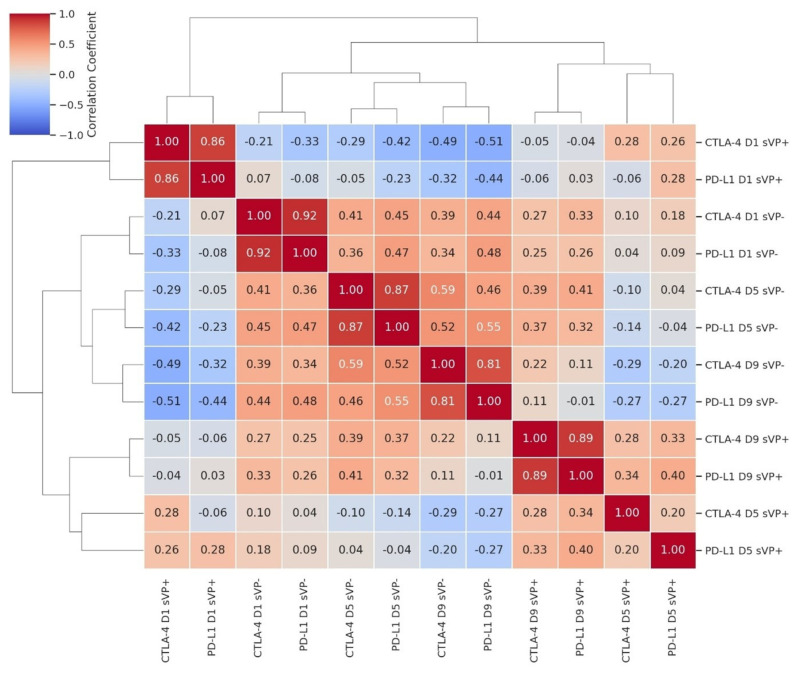
The clustered heatmap displays the Spearman’s correlation coefficients between serum levels of CTLA-4 and PD-L1 measured on days 1 (D1), 5 (D5), and 9 (D9) in patients with (sVP+) and without (sVP−) symptomatic vasospasm. Each cell represents a correlation coefficient, with the intensity of red indicating a strong positive correlation and blue indicating a strong negative correlation. The dendrograms suggest hierarchical clustering of variables with similar correlation patterns.

Hierarchical clustering of serum CTLA-4 and PD-L1 levels across D1, D5, and D9 revealed distinct patterns between patients with and without secondary vasospasm (sVP), Figure 2. In the sVP− group, all six measurements clustered tightly, indicating strong internal correlations. Similarly, the sVP+ group exhibited robust clustering, particularly at later timepoints (D5 and D9). In contrast, cross-group comparisons revealed weak or negative correlations. Notably, a temporal structure (D1 → D5 → D9) was apparent within each group, with a clearer trajectory in the sVP+ cluster, reflecting a time-dependent shift in immune modulation.

## 3. Discussion

This study provides the first evidence that elevated serum levels of sCTLA-4 and sPD-L1 are associated with the development of sVP following aSAH. Both immune checkpoint markers demonstrated a time-dependent increase from D1 to D9 post-ictus in sVP+ patients, implicating their role in ongoing immune activation. Significant differences in sCTLA-4 and sPD-L1 levels were observed between sVP+ and sVP− groups on D5 and D9, whereas no differences were found between sVP− patients and healthy controls. Hierarchical clustering revealed distinct and progressive immune signatures in sVP+ patients, further supporting a dynamic, vasospasm-specific immune response trajectory.

In the acute phase of aSAH, patients often exhibit systemic inflammatory response syndrome (SIRS), with its severity serving as a predictor of poor clinical outcomes [24]. Prolonged inflammation is further linked to complications such as cerebral vasospasm and delayed cerebral ischemia [25,26]. Early research observed that patients with aSAH who developed vasospasm exhibited elevated white blood cell counts, complement activation, and increased levels of circulating immune complexes compared to those without vasospasm [27,28,29,30]. Histopathological analyses further revealed infiltration of lymphocytes, macrophages, and granulocytes, along with immunoglobulin and complement deposits within the vessel walls [31,32]. In the acute phase, dying cells and breakdown products of blood release damage-associated molecular patterns (DAMPs), including S100 protein, heme, and oxyhemoglobin, which trigger widespread activation of Toll-like receptors (TLRs) [33,34]. Kim et al. highlights that PD-1 and CTLA-4 are upregulated in cerebrovascular injuries, including vasospasm after aSAH, where immune checkpoints attempt to regulate excessive inflammation [11]. Ongoing immune activation progresses into the chronic phase, which is primarily driven by B and T lymphocytes along with infiltrating macrophages. These immune cells can continue to produce inflammatory cytokines and reactive oxygen species (ROS), thereby amplifying the inflammatory response [11]. The increase in soluble checkpoint molecules (sCTLA-4 and sPD-L1) from days 1 to 9 in sVP+ patients in our study suggests a progressive immune response and checkpoint activation in response to ongoing inflammation and immune dysregulation following the hemorrhage. The observed differences in our study, particularly on D5 and D9, highlight a potential role for sCTLA-4 and sPD-L1 as biomarkers of subacute inflammation and vasospasm risk. This timing coincides with the peak of cerebral vasospasm, which typically occurs between days 3 and 14 after aSAH, reinforcing the clinical relevance of these biomarkers. Our findings are consistent with a prior mechanistic study [35] identifying PD-1+ monocytes as mediators of vasospasm, supporting the hypothesis that immune checkpoint pathways are actively involved in the pathogenesis of post-aSAH neuroinflammation. Other previous studies highlight the significance of immune checkpoint molecules, including CTLA-4 and Tim-3, in modulating inflammatory responses in intracranial aneurysms (IA). The observed dysregulation of these pathways in IA patients aligns with our findings of elevated sCTLA-4 and sPD-L1 levels in aSAH patients with symptomatic vasospasm, suggesting a broader role for immune checkpoint dysfunction in the pathogenesis of cerebrovascular inflammatory conditions [36,37,38]. Our observation that sVP+ patients display a unique immunologic signature via clustering analysis likely reflects differential immune activation pathways and supports the hypothesis that immune checkpoint modulation may be key in disease progression or recovery. PD-L1 administration was shown to prevent vasospasm in a murine model [35], and PD-1+ monocytes were identified as potential biomarkers and therapeutic targets in aSAH-related vasospasm. Similarly, CTLA-4 plays a regulatory role via Treg function [11], and its upregulation may reflect a homeostatic attempt to suppress inflammation that fails in sVP+ patients, leading to prolonged immune activation and worse outcomes.

Our results are further supported by Chaudhry et al. [39], who previously demonstrated increased frequencies of regulatory T cells (Tregs) and their activated subsets (HLA-DR− CD38+ and HLA-DR+ CD38+) in SAH patients, particularly during the delayed brain injury (DBI) phase and in those with post-aSAH complications such as cerebral vasospasm, seizures, and infections. Given that activated Tregs are known to express high levels of membrane-bound CTLA-4 and PD-L1, the elevated soluble levels observed in our study likely reflect the expansion and activation of this immunosuppressive T cell population in sVP+ patients. Additionally, their results highlight a disrupted Th17/Treg balance and identify correlations between specific Treg activation states and the occurrence of vasospasm. These findings reinforce the clinical relevance of immune checkpoint dysregulation in aSAH and provide mechanistic support for our observations, indicating that sCTLA-4 and sPD-L1 may serve as dynamic biomarkers of immune activation and vasospasm risk.

Our results also indicate that in the vasospasm-positive (sVP+) group, CTLA-4 and PD-L1 levels remain consistently elevated regardless of the presence or absence of infection (Table 3), supporting the interpretation that vasospasm itself is the primary driver of these changes. However, since the overall rate of infection was significantly higher in sVP+ patients (46% vs. 26%), we cannot fully exclude the possibility that systemic inflammatory responses, particularly affecting sPD-L1, contributed in part to the observed elevations. These findings therefore suggest a predominant but not exclusive role of vasospasm in shaping checkpoint dynamics. Nevertheless, it is equally plausible that these elevations represent a systemic consequence of symptomatic vasospasm-related inflammation rather than its direct cause. These findings imply that immune checkpoint activation in sVP+ patients reflect a vasospasm-specific inflammatory or immunoregulatory response, rather than being secondary to infectious complications. This interpretation is clinically important, as it supports the role of sCTLA-4 and sPD-L1 as biomarkers specifically linked to vasospasm, potentially independent of confounding inflammatory stimuli such as infection. Moreover, Feghali et al. [40] have recently reviewed therapeutic strategies targeting the PD-1 axis in cerebrovascular injury, including aSAH-induced vasospasm, highlighting the translational potential of checkpoint modulation in mitigating secondary injury.

Several other studies have confirmed the role of immune checkpoint molecules in other central nervous system pathologies. CTLA-4 signaling has been identified as a dominant immune pathway activated within 24–48 h following ischemic stroke, indicating its role in post-stroke immune suppression [41]. Elevated CTLA-4 levels have also been observed in hypertensive stroke patients and were integrated into a prognostic model that accurately predicts unfavorable clinical outcomes [42]. In parallel, soluble PD-L1 exerts a protective effect in stroke by activating the PD-1 pathway on circulating monocytes, which helps reduce neuroinflammation and brain edema and improves survival and recovery in animal models [43]. The PD-1 pathway also confers neuroprotection through its activity in microglia and macrophages [44]. Specifically, PD-1 signaling suppresses the pro-inflammatory M1 phenotype and promotes the anti-inflammatory M2 phenotype in these cells, contributing to reduced neuroinflammation after spinal cord injury [45]. Several studies suggest that PD-L1 can aggravate inflammation in stroke, with anti-PD-L1 antibody treatment helping to reduce CNS inflammation [46]. In contrast, other research shows that PD-L1 can alleviate neurological deficits and offer neuroprotective effects in stroke [47,48]. These conflicting findings highlight the dual and context-dependent role of PD-L1/PD-1 signaling in CNS inflammatory responses. Interestingly, while both sCTLA-4 and sPD-L1 increased in sVP+ patients, the relative dynamic change was greater for sPD-L1. This may be explained by their differing biological origins: sCTLA-4 is secreted primarily by activated T cells, reflecting regulatory T cell activity, whereas sPD-L1 can be released by multiple immune and vascular cell types through both alternative splicing and proteolytic shedding. Such broader cellular involvement may make sPD-L1 a more sensitive marker of systemic and cerebrovascular immune activation. Although PD-L1 signaling has been described as neuroprotective in ischemic stroke models, elevated circulating sPD-L1 may have different implications: soluble PD-L1 could act as a decoy receptor that impairs protective PD-1 signaling, or alternatively, it may simply reflect the magnitude of the inflammatory response. Therefore, high sPD-L1 levels in our study likely indicate an intensified systemic and vascular immune activation associated with vasospasm, rather than a direct protective effect. Collectively, these findings suggest that both CTLA-4 and PD-1/PD-L1 pathways play crucial roles in modulating post-stroke inflammation and recovery, offering potential targets for therapeutic intervention. Our findings also carry important translational implications beyond the field of cerebrovascular disease. The immune checkpoints CTLA-4 and PD-1/PD-L1 axis are currently central targets of cancer immunotherapy, where checkpoint inhibitors have revolutionized treatment by enhancing anti-tumor T-cell responses [49]. In contrast, our results suggest that in cerebrovascular injury, such as aSAH-related vasospasm, the pathological process is characterized by excessive immune activation rather than insufficient immunity. Thus, therapeutic strategies might require the opposite approach—namely checkpoint agonism or enhancement, rather than blockade—to dampen inflammation and protect the neurovascular unit. This complementary perspective illustrates how dynamic monitoring of soluble checkpoint molecules in aSAH patients can not only deepen our understanding of vasospasm-specific immune trajectories but may also provide key insights relevant for oncology by showing how checkpoint biology operates in a non-malignant but highly inflammatory context. As such, our study highlights the dual and context-dependent role of immune checkpoint pathways and provides a framework for future translational research at the intersection of neuroinflammation and cancer immunotherapy.

This study has several limitations that should be acknowledged. First, although the prospective design strengthens the reliability of our findings, the single-center setting may limit the generalizability of the results to broader or more diverse patient populations. Future multicenter studies with larger sample sizes are warranted to validate these findings across varied clinical settings. Second, while we excluded patients with overt infections at admission, the significantly higher rate of infections in the sVP+ group (46% vs. 26%) could have influenced immune marker levels, particularly sPD-L1, which is known to be elevated in systemic inflammatory states. More rigorous longitudinal monitoring for subclinical infections and adjustment for confounding inflammatory conditions in statistical models may help clarify this association.

Third, while CTLA-4 and PD-L1 were measured at three key time points (days 1, 5, and 9), additional sampling across the vasospasm window (days 3–14 post-ictus) might better capture temporal immune dynamics and improve the temporal resolution of biomarker kinetics. Fourth, the study did not assess cellular immune phenotypes or functional assays (e.g., T cell or monocyte checkpoint receptor expression), which could help link soluble biomarker levels to specific immune mechanisms. Integrating flow cytometry or single-cell analyses in future research could provide mechanistic insights and strengthen the biological plausibility of these markers.

Lastly, while we identified strong associations between elevated immune checkpoint levels and symptomatic vasospasm, causality cannot be inferred from this observational study. Experimental studies and interventional trials targeting checkpoint pathways will be essential to determine whether these molecules are viable therapeutic targets in aSAH.

## 4. Materials and Methods

### 4.1. Participants

This prospective study enrolled 179 patients diagnosed with aSAH at our institution between January 2022 and December 2024.

Eligible participants met the following inclusion criteria: (1) age over 18 years; (2) confirmed aSAH diagnosis by non-contrast head CT and aneurysm confirmed by CTA or DSA; and (3) presentation within 24 h of the ictus. Exclusion criteria included traumatic SAH, pregnancy, hospital admission beyond 24 h after symptom onset, lack of aneurysm treatment, bleeding from arteriovenous malformation, missing informed consent, significant systemic comorbidities (e.g., malignancy, liver/renal insufficiency, chronic lung disease, and inflammatory bowel or other chronic gastrointestinal diseases), chronic infections, signs of acute infection on admission, aneurysm rerupture, or clinical deterioration during treatment.

Comprehensive clinical data were recorded for all enrolled patients, including demographics (age and sex), vascular risk factors (hypertension, diabetes, and smoking status), admission laboratory values, WFNS and modified Fisher scores, intensive care interventions (e.g., mechanical ventilation and extraventricular or lumbar drainage), occurrence and type of infections, presence of symptomatic vasospasm, and 3-month clinical outcomes assessed by the modified Rankin Scale (mRS). Follow-up was conducted via telephone or in-person visits; a favorable outcome was defined as mRS 0–3 at 3 months.

The primary outcome was the association between immune checkpoint molecules sCTLA-4 and sPD-L1 in patients with aSAH and symptomatic vasospasm. Secondary outcomes included the relationship of these markers with favorable versus unfavorable outcomes. Comparisons were also made between aSAH patients and healthy controls to evaluate baseline marker differences.

Outcome assessments were performed by independent, trained, blinded evaluators not involved in patient care at 3 months (±5 days) post-aSAH. All patients received standard prophylactic treatment with nimodipine (60 mg orally every 4 h from day 1 to prevent vasospasm). Symptomatic vasospasm (sVP) was defined [50] as new neurological deficits that correlate with angiographic vasospasm in the absence of other causes. Diagnosis required consensus by at least two neurointensivists. Asymptomatic large vessel vasospasm without accompanying clinical or radiological findings was not classified as symptomatic vasospasm.

Systemic and central nervous system infections were defined by clinical signs (e.g., fever > 38 °C), elevated inflammatory markers (rising CRP or procalcitonin > 0.5 ng/mL), and a positive diagnostic test (e.g., chest X-ray, CSF or blood culture, or urine analysis).

The control group (*n* = 50) consisted of patients with radiologically confirmed cerebral aneurysms who were not treated due to low rupture risk or personal preference. In these individuals, serum sampling was conducted following confirmation of eligibility and exclusion of ongoing infections, verified through laboratory testing to avoid confounding marker levels.

### 4.2. Standard Protocol Approvals and Patient Consent

Institutional review board approval was obtained prior to study initiation (IV/8468-1/2021/EKU, 27 October 2021 and BM/4629-1/2024), and written informed consent was collected from all participants or their legal representatives before inclusion.

### 4.3. Serum CTLA-4 and PD-L1 Analysis

The concentration of PD-L1 and CTLA-4 was determined as described previously by our group with minor modifications [51,52]. Arterial blood samples were collected from all eligible patients on the first day (D1), fifth day (D5) after ictus, and ninth day (D9) following the ictus. Briefly, the serum fractions were stored at −80 °C in aliquots. To remove debris, serum samples were centrifuged at 10,000× *g* for 5 min at 4 °C before running the assay. The concentration of the following immune-oncology checkpoint proteins was determined using the Luminex xMAP (MAGPIX^®^, Luminex Molecular Diagnostics Inc., Austin, TX, USA) technology according to the instructions of the manufacturer: PD-L1 and CTLA-4 (MILLIPLEX^®^ Human Immuno-Oncology Checkpoint Protein Panel 1, Cat: HCKP1-11K, Merck, Rahway, NJ, USA). All samples were thawed and tested in a blind fashion. Serial dilution of the recombinant standards was performed following the instructions of the manufacturer. The plate was washed with 200 μL wash buffer, and 25 μL of standard or quality control (QC) samples were added to the appropriate wells. In addition, 25 μL of matrix solution was added into the standard, background, and QC wells. Lyophilized serum matrix solution provides a more accurate analyte quantification than a standard curve diluted in buffer; serum matrix simulates the conditions in which the native protein is analyzed. QC1 and QC2 samples were applied as internal/positive controls provided by the manufacturer. Recombinant proteins at two dilutions were run in repeat assays to establish a target QC range, which was used to verify assay performance compared to kit specifications. Twenty-five μL of assay buffer was added to background wells, 50 μL of each undiluted serum sample was added to the appropriate wells, and 25 μL of capture antibody-coated, fluorescent-coded Luminex beads was added to all wells. The final volume of each reaction was 75 μL in one well. The plate was incubated overnight (16 h) at 4 °C. The plate was washed three times with 200 μL wash buffer. Next, 25 μL biotinylated detection antibody mixture was added to each well and incubated for 1 h at RT (room temperature), then 25 μL Streptavidin-PE was added to each well and incubated 30 min at RT. After the last washing step, 150 μL Drive Fluid Plus was added to each well and the plate was incubated for an additional 5 min on a plate shaker and read on the Luminex MAGPIX^®^ instrument. Luminex xPonent 4.2 software was used for data acquisition. Five-PL regression curves were generated to plot the standard curves for all analytes by the Belysa Immunoassay Curve-Fitting software (Version 1.2) calculating with bead median fluorescence intensity values. The detection range for PD-L1 was between 1.1 and 20,000 pg/mL; for CTLA4, it was between 1.3 and 50,000 pg/mL.

The soluble form of CTLA-4 and PD-L1 will be referred to as CTLA-4 and PD-L1 in the following text.

### 4.4. Statistical Analysis

Statistical analysis was performed using GraphPad Prism (version 10.0.2) and SPSS (version 25). Continuous variables were expressed as means with standard deviations (SD) for normally distributed data or medians with interquartile ranges (IQR) for non-normally distributed data. Group comparisons for continuous variables were performed using the independent samples *t*-test or the Mann–Whitney U test, depending on data distribution. Categorical variables were compared using the chi-square test or Fisher’s exact test where appropriate. To evaluate differences in serum levels of CTLA-4 and PD-L1 between groups (control vs. aSAH and sVP− vs. sVP+), the Mann–Whitney U test was used. Changes in immune checkpoint levels over time (days 1, 5, and 9) were analyzed separately for the sVP− and sVP+ groups using nonparametric comparisons. Correlations between CTLA-4 and PD-L1 at different time points were assessed using Spearman correlation coefficients. A clustered heatmap was generated to visualize correlation patterns, and hierarchical clustering was applied to identify similarity structures among variables. A *p*-value of <0.05 was considered statistically significant. Significance levels in figures were indicated as follows: * *p* < 0.05, ** *p* < 0.01, *** *p* < 0.001, and **** *p* < 0.0001.

### 4.5. Data Availability

Anonymized data not published within this article will be made available by request from any qualified investigator.

## 5. Conclusions

This study provides novel evidence that elevated serum levels of soluble CTLA-4 and PD-L1 are associated with the development of symptomatic vasospasm following aneurysmal subarachnoid hemorrhage. Both markers showed a time-dependent increase in sVP+ patients, with significant differences emerging on days 5 and 9, suggesting a sustained immune activation linked to vasospasm risk. Our findings align with previous mechanistic studies demonstrating upregulation of immune checkpoints in cerebrovascular injury and the involvement of activated regulatory T cells in post-SAH complications. These results support the potential of sCTLA-4 and sPD-L1 as dynamic biomarkers for vasospasm and underscore the relevance of immune checkpoint pathways in cerebrovascular inflammation. Future studies are needed to validate these biomarkers and explore their therapeutic implications in modulating immune responses after aSAH.

## Figures and Tables

**Table 1:** Clinical characteristics of study participants.

Variable	Total (*n* = 179)	sVP− (*n* = 131)	sVP+ (*n* = 48)	*p*-Value
Age (mean ± SD)	57.5 ± 12	58 ± 13	56 ± 12	0.374
Female, N (%)	130 (72)	93 (71)	37 (76)	0.597
Hypertension, N (%)	95 (53)	67 (51)	28 (58)	0.432
Diabetes, N (%)	18 (10)	11 (8)	6 (13)	0.425
Smoking, N (%)	52 (29)	39 (30)	13 (27)	0.863
WFNS, median (IQR)	2 (1–4)	2 (1–4)	2 (1–5)	0.673
mFisher score, median (IQR)	3 (2–3)	3 (2–3.5)	3 (2–3)	0.290
Loss of consciousness during ictus, N (%)	68 (38)	50 (38)	18 (38)	1.000
Seizure during ictus, N (%)	16 (9)	11 (9)	5 (11)	0.595
Aneurysm location, N (%)				
internal carotid artery	15 (9)	10 (8)	5 (11)	
middle cerebral artery	51 (28)	39 (30)	12 (25)	
anterior communicating artery	61 (34)	42 (33)	19 (39)	
posterior communicating artery	20 (11)	15 (12)	5 (10)	
anterior cerebral artery	7 (4)	5 (4)	2 (5)	
vertebrobasilar	25 (14)	20 (15)	5 (10)	
C-reactive protein ^a^, mg/L, median (IQR)	15.3 (4–55)	12 (4–60)	22 (3–53)	0.550
Creatinine ^a^, µmol/L, median (IQR)	60 (49–72)	61 (51–74)	59 (43–70)	0.100
White blood cell count ^a^, G/L, median (IQR)	11 (9–13)	11 (9–13)	11 (9–15)	0.317
Neutrophile-lymphocyte ratio ^a^, median (IQR)	5.5 (4–10)	5.4 (3–10)	6.5 (4–10)	0.271
Lumbal drain, N (%)	90 (50)	61 (47)	29 (62)	0.083
Mechanical ventillation, N (%)	81 (45)	58 (44)	23 (49)	0.532
Decompressive craniotomy, N (%)	18 (10)	14 (11)	4 (9)	0.802
Extraventricular drainage, N (%)	81 (46)	56 (43)	25 (51)	0.346
Infection, N (%)	56 (31)	34 (26)	22 (46)	0.011

SD, standard deviation, N, number, WFNS, World Federation of Neurological Societies, IQR, interquartile range, ^a^ on admission, we defined favorable outcomes as modified Rankin score (mRS) 0–3, while unfavorable outcomes were determined as mRS 4–6.

**Table 2:** Comparison of CTLA-4 and PD-L1 Levels at days 1, 5, and 9 between patients with and without symptomatic vasospasm (sVP) and healthy controls (ctrl).

pg/mL	sVP− (*n* = 131) Median (IQR)	sVP+ (*n* = 48) Median (IQR)	Ctrl (*n* = 50) Median (IQR)	*p*-Value (sVP− vs. sVP+)	*p*-Value (sVP− vs. Ctrl)	*p*-Value (sVP+ vs. Ctrl)
CTLA-4-D1	3 (0–9.6)	4.6 (1–12.5)	0 (0–3.1)	NS	0.073	0.002
CTLA-4-D5	0.1 (0–5.1)	4.6 (0.3–10.6)	0 (0–3.1)	0.001	NS	0.001
CTLA-4-D9	1 (0–4.5)	5.6 (1–12.7)	0 (0–3.1)	<0.001	NS	<0.001
PD-L1-D1	24.5 (12.7–51.1)	33.2 (19.4–54.2)	12.5 (3.6–31.5)	NS	0.006	<0.001
PD-L1-D5	19.7 (12.5–33.9)	37.3 (17.6–55.4)	12.5 (3.6–31.5)	0.001	NS	<0.001
PD-L1-D9	20.7 (12.1–34.1)	41.4 (19.3–67)	12.5 (3.6–31.5)	<0.001	NS	<0.001

CTLA-4, cytotoxic T-lymphocyte-associated protein 4; PD-L1, programmed death-ligand 1; sVP, symptomatic vasospasm; ctrl, control; D1/D5/D9, days 1, 5, and 9 post-ictus; NS, not significant.

**Table 3:** Comparison of sCTLA-4 and sPD-L1 Levels on days 5 and 9 in vasospasm-positive and vasospasm-negative aSAH patients with and without infection.

pg/mL	sVP−/Inf−	sVP−/Inf+	sVP+/Inf−	sVP+/Inf+	*p*-Value (sVP+/Inf− vs. sVP+/Inf+)
CTLA4D5	0.73	0.00	5.29	2.48	NS
CTLA4D9	1.48	0.01	4.11	6.41	NS
PDL1D5	19.86	16.59	38.29	37.30	NS
PDL1D9	22.49	17.61	41.52	40.57	NS

Values represent median serum concentrations of soluble CTLA-4 (sCTLA-4) and soluble PD-L1 (sPD-L1) measured on day 5 (D5) and day 9 (D9) post-ictus. Data are stratified by vasospasm status (sVP+/sVP−) and infection status (Inf+/Inf−). aSAH—aneurysmal subarachnoid hemorrhage; sVP—symptomatic vasospasm; sCTLA-4—soluble cytotoxic T-lymphocyte-associated protein 4; sPD-L1—soluble programmed death-ligand 1; D5—day 5 post-ictus; D9—day 9 post-ictus. NS, not significant.

## Data Availability

Anonymized data not published within this article will be made available by request from any qualified investigator.

## Abbreviations

The following abbreviations are used in this manuscript:

aSAH	Aneurysmal Subarachnoid Hemorrhage
IA	Intracranial Aneurysm
DCI	Delayed Cerebral Ischemia
CTLA-4	Cytotoxic T-lymphocyte Antigen-4
PD-L1	Programmed Death-Ligand 1
sCTLA-4	Soluble Cytotoxic T-lymphocyte Antigen-4
sPD-L1	Soluble Programmed Death-Ligand 1
mPD-L1	Membrane-bound Programmed Death-Ligand 1
exoPD-L1	Exosomal Programmed Death-Ligand 1
MMP	Matrix Metalloproteinase
TLR	Toll-like Receptor
DAMP	Damage-Associated Molecular Pattern
SIRS	Systemic Inflammatory Response Syndrome
Treg	Regulatory T Cell
Th17	T-helper 17 Cell
WFNS	World Federation of Neurological Societies
mRS	Modified Rankin Scale
CTA	Computed Tomography Angiography
DSA	Digital Subtraction Angiography
AVM	Arteriovenous Malformation
CRP	C-Reactive Protein
SD	Standard Deviation
IQR	Interquartile Range
NSCLC	Non-Small Cell Lung Cancer
CNS	Central Nervous System
ROS	Reactive Oxygen Species
HLA-DR	Human Leukocyte Antigen-DR isotype
